# Dependency Factors in Evidence Theory: An Analysis in an Information Fusion Scenario Applied in Adverse Drug Reactions

**DOI:** 10.3390/s22062310

**Published:** 2022-03-16

**Authors:** Luiz Alberto Pereira Afonso Ribeiro, Ana Cristina Bicharra Garcia, Paulo Sérgio Medeiros dos Santos

**Affiliations:** PPGI-Informatics Department, UNIRIO Universidade Federal do Estado do Rio de Janeiro, Rio de Janeiro 22290-240, Brazil; cristina.bicharra@uniriotec.br (A.C.B.G.); pasemes@uniriotec.br (P.S.M.d.S.)

**Keywords:** information fusion, Dempster–Shafer Theory, Bayesian network, electronic health records, machine learning, adverse drug reactions

## Abstract

Multisensor information fusion brings challenges such as data heterogeneity, source precision, and the merger of uncertainties that impact the quality of classifiers. A widely used approach for classification problems in a multisensor context is the Dempster–Shafer Theory. This approach considers the beliefs attached to each source to consolidate the information concerning the hypotheses to come up with a classifier with higher precision. Nevertheless, the fundamental premise for using the approach is that sources are independent and that the classification hypotheses are mutually exclusive. Some approaches ignore this premise, which can lead to unreliable results. There are other approaches, based on statistics and machine learning techniques, that expurgate the dependencies or include a discount factor to mitigate the risk of dependencies. We propose a novel approach based on Bayesian net, Pearson’s test, and linear regression to adjust the beliefs for more accurate data fusion, mitigating possible correlations or dependencies. We tested our approach by applying it in the domain of adverse drug reactions discovery. The experiment used nine databases containing data from 50,000 active patients of a Brazilian cancer hospital, including clinical exams, laboratory tests, physicians’ anamnesis, medical prescriptions, clinical notes, medicine leaflets packages, international classification of disease, and sickness diagnosis models. This study had the hospital’s ethical committee approval. A statistically significant improvement in the precision and recall of the results was obtained compared with existing approaches. The results obtained show that the credibility index proposed by the model significantly increases the quality of the evidence generated with the algorithm Random Forest. A benchmark was performed between three datasets, incremented gradually with attributes of a credibility index, obtaining a precision of 92%. Finally, we performed a benchmark with a public base of heart disease, achieving good results.

## 1. Introduction

The better you know about a problem, the better you decide what to do about it. Data are largely available in current days, usually in varied formats, multiple sources, and multiple modes. The Internet of Things connects more than 9 billion devices, including smart things, enabling new interactions and interoperations between things and people [1]. In a heavily digitized world in which multiple sensors collect data from the environment, it is possible to integrate a large amount of information to generate knowledge. However, it is necessary to deal with doubts, inaccuracies, events with low chances of occurrence, and outliers, regardless of the domain. Together these aspects represent uncertainties that must be appropriately considered when making sense of data so that decision-making can be reliable.

All of these issues are within the scope of Data Fusion, defined in [2] as the integration of data and knowledge from multiple sources. In Multisensor Information Fusion (MSIF), data from different sensors are combined to provide a complete description of a situation of interest [3]. MSIF allows for decisions to be made with a more accurate understanding of the context and with lower uncertainty [1]. According to [4], MSIF provides better data analysis by (a) increasing the accuracy of information using various data sources, (b) mitigating uncertainty to provide an improved foundation for decision making, and (c) reducing the dimensionality of relevant data to provide an easily understandable view of a domain. MSIF faces several challenges in structural information due to data and sensors issues; data can suffer from conflicts and correlations; and sensors impose technical difficulties such as inaccuracies, frequency of capture, and redundancy [2,5,6].

The complexity of data fusion is caused by the diversity of types of data and collection environment and by the temporal dependencies among facts and evidence.

Furthermore, real-time applications involve data that have a meaningful effect only for a limited period. It is the challenge of dynamicity, where eventual occurrences last for a limited time. Distinguishing the proper order of facts is crucial for extracting the precise meaning of the information [7].

The current approaches to dealing with the challenge of uncertainty resolution during data fusion are fuzzy reasoning, Bayesian probability theory, and Dempster–Shafer Theory (DST) [8]. The first, Fuzzy reasoning, is a computation-based approach that mimics human reasoning, defining functions or membership grades that produce intermediate values possibilities. Logical fuzzy can be embedded in hardware and is widely used in control and artificial intelligence systems. The second, probability theory, contains two probability interpretations: the Bayesian and the frequentist. The frequentist approach only uses data, while Bayesian reasoning uses data to augment the previous belief. By combining data and an initial probability, the model benefits from a fair amount of prior knowledge is available [9]. The third, DST, was created to handle uncertainty and incomplete information; to allow partitions to evidence a combination from different sources; and to compute a degree of belief, called a belief function, that considers all available evidence [10]. DST is a reasoning widely used in fault diagnosis, medical diagnosis, decision systems, and risk analysis. Unlike the Bayesian framework, DST allows for the assignment of belief to sets of elements in the domain. On the other hand, the Bayesian model assigns beliefs only to individual elements. In our work, we use a Bayesian network to perform inferences that support beliefs in a DST context.

DST was designed to deal with conflict between expert opinions. The opinions must be independent and come from different sources without any dependency [10]. We carried out a literature review that identified a growing interest in the use of DST in MSIF, mainly because of the integration of MSIF with machine learning techniques. This integration reinforces the importance of estimating beliefs to use as input to the Basic Probability Assignment Function (BPA) from data sets. Consequently, a robust statistical treatment is essential in the pre-processing of MSIF models with DST to improve the quality of BPA generation, whether in the use of probabilistic contribution methods in [11] or approaches with machine learning [12]. We also observed in the literature review the misuse of data analysis on the dependence between sources and sensors, highlighting the importance of analyzing these relationships [13]. Therefore, considering these assumptions, it is necessary to propose a new way to apply the MSIF with DST.

To further understand the uncertainty issue, let us suppose a fictional example in which uncertainty is present but is not readily perceptible. Mary went to the hospital as she was experiencing abdominal pain and complaining of digestive bleeding. There were two doctors on duty: John and Paul. Mary is a patient with gynecologic cancer in an advanced stage that can present genital but not digestive bleeding. Since the last medical evaluation, she has been complaining of abdominal pain. She has been taking strong anti-inflammatory medication for a month and returned to the hospital with a bit of digestive bleeding. John and Paul initially discussed the case. John considers digestive bleeding to be due to the anti-inflammatory medicine, with an 80% chance, attributing another 20% chance to something else. Paul disagrees with John. Paul believes, with 70% chance, that the digestive bleeding was caused by cancer’s natural evolution. He did not eliminate an adverse drug reaction (ADR) to the anti-inflammatory medicine but only attributes a 20% chance to this being true. In addition, he considers that there is a 10% chance of the cause being something else. These are two conflicting opinions for a diagnosis. This type of discussion is a classic DST dilemma.

Both experts agreed that more investigation was needed and decided to perform a deeper study, looking at clinical notes, the latest medical prescriptions, and a detailed pharmacological study considering drug interactions. In addition, they requested the technicians to prepare (i) an analysis of all of the medication prescribed to the patient, (ii) the correlation between these drugs and ADR, (iii) another analysis with the possibilities of disease evolution, and (iv) an analysis of the possible trends in the blood parameters in laboratory tests last year. Finally, the specialists asked the staff for help making inferences with conditional probabilities related to the symptoms and the patient’s disease, and they asked for a statistical analysis about the drugs prescribed and ADR possibility.

Something caught their attention when analyzing the evolution of a liver enzyme in the last three exams. When they examined it in more detail, they identified a scenario of metastasis to the liver.

The variation-shown growth trend of the value of this enzyme established a relationship of dependence with the evolution of the disease. The increase in the liver enzyme was the causal factor that determined the metastasis of the disease to another organ. An increase in this enzyme most likely caused digestive bleeding. This phenomenon is a symptom of the disease’s metastasis to the liver. It virtually eliminates or substantially reduces the belief in an adverse effect of the anti-inflammatory medicine. Unfortunately, John and Paul did not have much time, and there was no way to organize the data quickly. The experts John and Paul were unable to obtain comparative studies with a historical analysis and to evaluate ADR in relation to cases of disease evolution in patients with that type of diagnosis. After all this effort, both specialists reached a consensus on the digestive bleeding. However, the anti-inflammatory drug possibly potentiating such symptoms would have been a more plausible hypothesis for the diagnosis of disease progression to the liver, strongly reducing the belief in an ADR episode.

John and Paul’s hypotheses changed, with John attributing 10% chance to anti-inflammatory ADR and with Paul attributing 90% chance to disease evolution.

This example demonstrates that an adverse reaction occurring in one patient, with a similar case, may not always happen in another. We are different biopsychosocial beings [14], and evidence used to identify ADR is influenced by several dependent and detailed aspects.

We were inspired by how experts solve their conflicts concerning their beliefs and based on the premise that identifying dependencies in a causal and temporal context contributes to adjusting uncertainties of belief functions. This study aims to propose a model of generation of BPA for DST in MSIF, mitigating the uncertainties caused by relationships dependency between data and sensors.

The hypothesis is that better results in terms of precision and less uncertainty are achieved when the impacts of dependency relationships from data in an MSIF environment are appropriately identified. Our contribution is the construction of a model using Bayesian inference, correlation, and temporal analysis to mitigate dependency conflicts arising from a dataset and the creation of a standardized method that defines a credibility index designed to generate evidence with improved precision.

The results obtained by implementing the model show that the proposed credibility index significantly increases the quality of evidence generated from the Random Forest technique. We perform a benchmark between three datasets, one with frequentist probability (base method), another with a credibility index added to the base method, and a third adding Bayesian inference to the second dataset. The second set of data allowed for a gain of 15% and the third had a precision of 21% greater. A public heart basis was used as a benchmark with satisfactory results.

The model was applied in the domain of ADR, where diagnostic uncertainty prevails, achieving significantly better accuracy and recall in an experiment that used electronic health records (EHR) data collected from a public hospital. These EHR gathered more than 50,000 active patients and 900,000 clinical records with 2 million drugs prescribed in 2019, where 63,000 ADR possibilities were evidenced in clinical notes involving 5937 patients.

The remainder of this paper is organized as follows. Section 2 presents the related work. Section 3 discusses background knowledge. Section 4 presents our proposal, material, and contributions of our work. Section 5 details the experiment. Section 6 presents the results. Section 7 presents a discussion. Finally, concluding remarks are presented.

## 2. Related Work

We present an overview of the main studies developed to deal with uncertainties due to dependencies. We note an emphasis on the use of the Bayesian net. Several theoretical methods were used, such as probabilistic, fuzzy, and Bayesian approaches. Our research contributes to creating a method that uses temporal and probabilistic dependence relationships. The model is in the context of multisensor sources and interaction between factors. Next, we cover the five related works.

The study in [15] identified conditionality and dependency between data sources, which used the Bayesian network with DST, creating an evidence network. This method used a priori knowledge made possible by using Bayesian networks. The advantages of this method are identifying the conditionalities and avoiding the inaccuracies in functions of mass and consequently belief functions. This study did not work with temporal interactions between required factors.

A new method used to deal with dependence on evidence in DST and divided into two steps was proposed in [13]. Initially, the relative weights of components in the system were quantified, considering their interactions. In a second step, the external dependency is described, using fractals using resources of pygnistic transformation of probability. Through sensitivity analyses, the proposal’s advantages in dealing with dependent evidence were demonstrated, improving the assessment of information loss. The downside is that it did not deal with BPA dependencies between hypotheses, only between sources.

The study in [16] proposed a method of combining dependent evidence based on mutual information between sources. To measure the degree of dependence between evidence, a matrix was generated for the discount coefficient to adjust the hypotheses’ uncertainties to match the Dempster rule. The results obtained were accuracies between 92 and 94% with the IRIS dataset, using the close correlation from the Pearson and Kendall methods. The advantage of the proposed method is that the correlation can deal with linear cases and non-linear cases that are more common in reality. The disadvantage is it does not implement causal inference.

The work is [17] used a Bayesian network structure and proposed a non-deterministic dependency analysis method that uses affine arithmetic. The advantage can produce a *p-box* of more restricted resulting reliability than those obtained by Frechet inequalities and is more economical than the *2-stage Monte Carlo method*. The downside is that it does not consider causality, temporality, correlation, and interactions, restricting its use to more complex problems.

Creating a retractable combination method that acts on a network of evidence that forms a DST discernment framework, the research [18] produces a coefficient matrix and adjusts the old belief functions. The advantage is that adjusting belief values sometimes leads to negative belief values that reverse the effect of the unwanted combination of evidence. When regular evidence is combined, negative belief values are used to influence belief values oppositely. The disadvantages are that it does not work with causality, temporality, or interactions and does not determine the degree of dependence on BPA. A Bayesian hierarchical multi-model approach was proposed in [19] to quantify the uncertainty in the form of dependency model and model parameters that result from small data sets. This approach identified a set of marginal candidates models and their corresponding model probabilities using the Bayesian inference model. Then, it estimated the uncertainty in a dependency structure based on the conditional copula technical on the marginals and their parameters. The model created a new sampling re-weighting approach to propagate uncertainties efficiently. It starts with a p-box-based probabilistic model; then, performed a linear approach for a non-deterministic dependency Bayesian network; and compared the results with Frechet inequality. The advantage was that it estimated the uncertainty in the amount of interest given the candidate multiple model input distributions at a low computational cost compared with typical nested Monte Carlo simulations. The downside was its computational complexity.

These work identify dependence in different ways but do not integrate the causal, correlational, and temporal dimensions and interaction between factors. The use of Bayesian inference together with DST is a relevant aspect. The correlation between factors was explored in greater depth in a few studies, and in particular, temporal dependence was not often investigated, possibly due to the need for a longitudinal study. The model addresses the aspects of interaction, correlation, causal and temporal dependence, integrating them in a credibility index to generate evidence in a more precise way.

## 3. Background Knowledge

In this section, we address the main theoretical foundations that we use in our proposal. They are MSIF, Bayesian Net, and DST.

### 3.1. MSIF Multisensor Information Fusion

MSIF is based on a tailored tier process involving aggregation, data mining, and integration of information from multiple sources, resulting in an information product of greater value than any single piece [20].

MSIF favors a cross-vision, not only of the data but also in the presentation, in the visual method. A multi-view-based data fusion method studies an object from the knowledge of different points of view [21].

This study follows an MSIF architecture model proposed by the data fusion information group (DFIG), prioritizing information management and user participation. DFIG advances the evolution of the Joint Defense Laboratory (JDL) seminal model and advocates for the application of MSIF in real-world management domains beyond the fields of robotics or military automation, using evidence-based reasoning methods to highlight DST [20].

**Figure 1:**
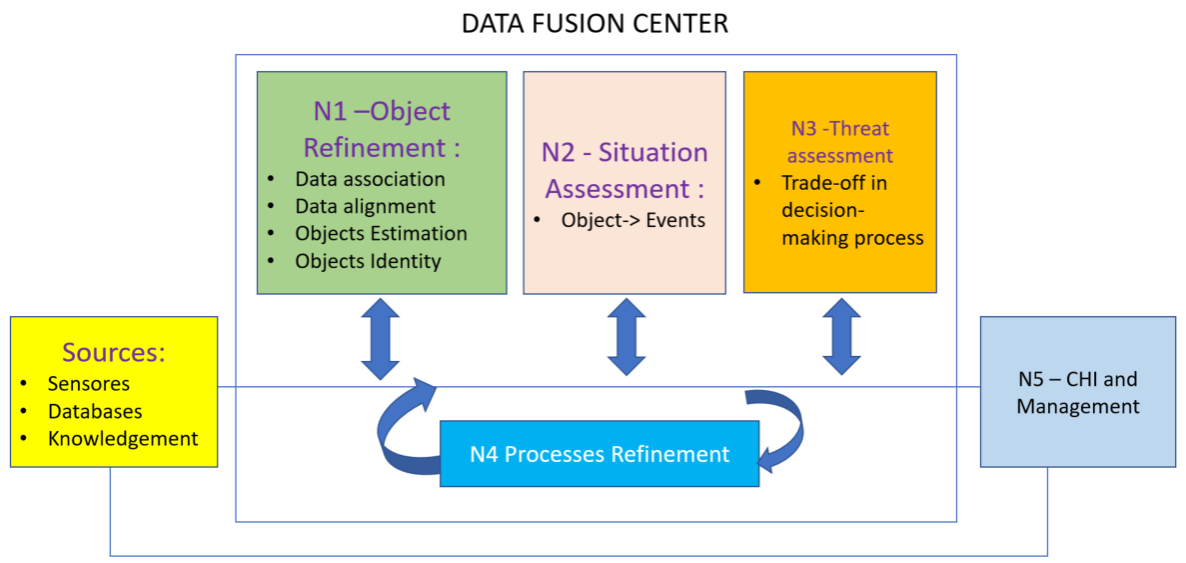
Data fusion center-model DFIG adapted [20].

Figure 1 shows the levels of fusion stages used in the proposed model. Each level has its function, contribution, and interaction and feedback between levels occurs, providing dynamism to the process.

The MSIF architecture is divided into six levels:

- Level 1 emphasizes the association between data, making it possible to identify and locate objects from properties and attributes. The combination of data occurs to obtain the most reliable and accurate estimate of an entity of interest’s position. The place and frequency of the objects are determined from the multiple sensors in the domain. Level 1 performs four basic operations, fundamental to the rest of the process, containing alignment, association, correlation, and classification procedures.
- Level 2 is the refinement of the situation. Level 2 uses data aggregation techniques and NLP for extracting data of interest from the domain.
- Level 3 is an impact assessment The a priori knowledge established in the previous levels is interpreted concerning the prediction of estimates and effects.
- Level 4 is process refinement. At this level, the goal of refinement processes is to close the model cycle. This level enables potential sources of enhanced information.
- Level 5 is object refinement. Finally, Level 5 allows for management of the model and the human–computer interaction (HCI), for the selection of domain objects, and for the specification of visual methods of presenting the results.

### 3.2. Bayesian Net

According to the definition of [22,23], the Bayesian Networks (BN) is an effective tool of Bayesian inference capable of representing uncertain and incomplete findings, based on conditional probability and not on Bayes’ Theorem. The Bayesian reasoning allows for defining the effects of causality among several in a system. A Directed Acyclic Graph (DAG) expresses a relationship of causality between variables. For discrete variations or knowledge, it is represented using conditional probability tables. For several continuous variations, probability density functions are used. Some of the main characteristics of a BN are satisfactory to the local Markov property, in which a variable is conditionally dependent only on its context. Assuming this premise, the rule chain used to calculate the joint probability of a variable can be simplified to the following:

$$P\left(X_{1}\ldots X_{n}\right)=\prod_{i=1}^{n}=P\left(X_{i}|parents\left(X_{i}\right)\right)$$

where $X_{i}$ is a random variable in BN and *parents* ($X_{i}$) is a set of all those that influence the variables.

### 3.3. Dempster–Shafer Theory

In DST, there is a fixed set of N mutually exclusive and exhaustive elements, called Frame of Discernment. Consider a set, indicated by

$$\Theta =\{\Theta_{1},\Theta_{2},\ldots \Theta_{i},\ldots ,\Theta_{N}\}.$$

The set Θ, represented by P(Θ), consists of all subsets of Θ. This includes ⌀, the empty set; P(Θ),⌀; and Θ itself, the $2^{N}$ composite set of Θ elements:

$$P\left(\Theta \right)=\{⌀,\Theta_{1},\ldots ,\Theta_{N},\left\{\Theta_{1},\Theta_{2}\right\},\ldots ,\left\{\Theta_{1},\Theta_{2},\ldots \Theta_{i}\right\},\ldots ,\Theta \}.$$

Considering element *A* of P(Θ), *m*(*A*) represents how strongly the evidence supports *A*. When *m*(*A*) > 0, *A* is called the focal element of the mass function. Given the piece of evidence, the mass assigned to each element of *P*(Θ) is equivalent to an indicative value of the belief assigned to it. DST defines this function as mass *m*, called BPA, which has the following properties:

P(Θ)→[0,1],A→[m(A)],

meeting the following conditions:

(1) $$m:2^{\Theta }\to \left[0,1\right]\;$$

(2) m(⌀)=0

(3) ∑m(A)=1

- Equation (1) Indicates that all subsets of Θ are assigned a belief value between 0 and 1;
- Equation (2) Indicates that a belief deposited in the empty set is always zero; and
- Equation (3) This property indicates that the sum of all assigned values must be one.

In DST, a mass function is also called BPA. Suppose that two BPAs operate in two sets of propositions B and C, indicated by $m_{1}⊕m_{2}$, respectively; they are combined by the orthogonal sum implemented in the Dempster Rule.

DST does not require a complete probability model to work and uses a set of hypotheses rather than segregated hypotheses. Based on the accumulation of evidence, DST aims to facilitate the attribution of the probability of belief (BPA) to hypotheses.

Based on the dynamic framework, which accepts changes, the reasoning is established, considering the impact of the collected evidence.

The impact of evidence in the original individual hypotheses and their groupings should be observed. Such groupings are still considered hypotheses because they are the subsets of Θ. The mass function m is similar to the probability density function of probability theory. However, such a function does not contain the Bayesian restriction that the sum of belief attributed to unit subsets (original hypotheses or singletons) has to be one. Therefore, confirming a specific belief for a singleton does not imply confirming the remaining belief for its denial. Thus, *m* would behave as a probability density function, in case m assigns values greater than zero to the subsets of units of Θ.

A focal element is the entire subset of the frame of discernment in which belief assignment mass is greater than zero, that is, *A*∈Θ for which *m*(*A*) > 0. The degree of belief in an *A* element of *P*(Θ) is written as Bel(A). This degree represents the minimum belief in hypothesis *A* due to the evidence. Therefore, it is defined as the sum of the BPAs made to all subsets of *A*:

$$\forall A|A\in P\left(\Theta \right):Bel\left(A\right)=\sum_{x\subseteq A}m\left(X\right)$$

## 4. DEP-DST: Model for Dependence Adjustment in DST

This section describes the DEP-DST approach in three subsections. First, we describe the architectural model and component variables to integrate a CI. Next, we describe the materials and experimental methods that implemented the model in the ADR domain, and finally, we detail the MSIF process. This proposal presents a model that addresses the problem of dependency relationships, identifying them using correlational, causal, and temporal analysis techniques and integrating them in the proposition of a method that generates a credibility index for use in the generation of beliefs in a DST context in MSIF.

### 4.1. Architectural Model

**Figure 2:**
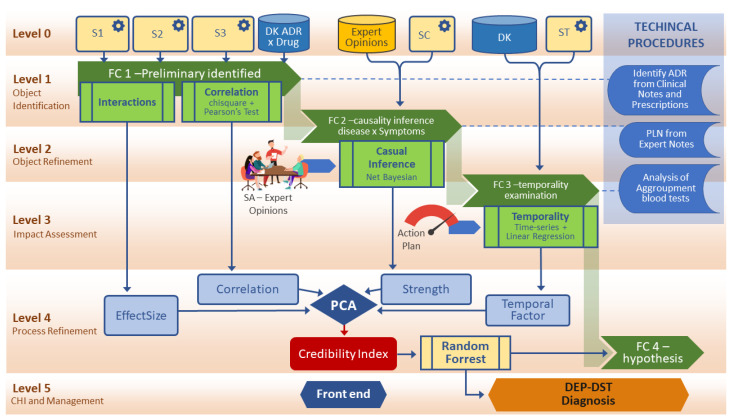
DEP-DST Model for dependence adjusts in DST.

Figure 2 describes the model created based on an MSIF process. The steps of the data fusion process contribute to creating the credibility index. As seen in DFIG model in Section 3.1, level 0 takes care of the pre-processing and organization of the capture. Identifying the level 1 object generates the fusion center 1, with the association of the primary data sources and sensors, allowing us to find the interactions between domain elements that allow for the generation of the size-effect variable. A correlation analysis is also feasible and enables the DepCorrelation variable to be used.

Based on the fusion center created at level 1, at level 2, the participation of experts is necessary for an assessment of the situation (SA) concerning the identification of the object, refining and adjusting details of the first level integration. These adjustments based on expert opinions generate evidence in a natural language format, requiring NLP procedures and data aggregation procedures that enable new sources of information. Potential objects of interest, discovered with the support of causal inference procedures, are identified, reaching the fusion center two.

Level 3 deals with impact assessment, which brings new possibilities for identifying objects of interest with the support of procedures planned in an action plan. At this point in the model, the processes that determine dependencies clarified by temporal effects are executed, and the temporality variable is generated to compose the CI.

Level 4 performs the refinement of the process as a whole. The estimation of the probability of DST is at this level, where the fusion center four is generated, which is the most robust base of the process. There is an interaction with the previous levels at this level, allowing for a review of procedures. The generation of BPA by the artificial intelligence method enables the generation of hypotheses for the decision-making DEP-DST model. Level 5 is for managing new requirements and goals of the MSIF process and user interface, and the visual methods.

### 4.2. Credibility Index—Integrating Components to Cleanup Dependencies

**Figure 3:**
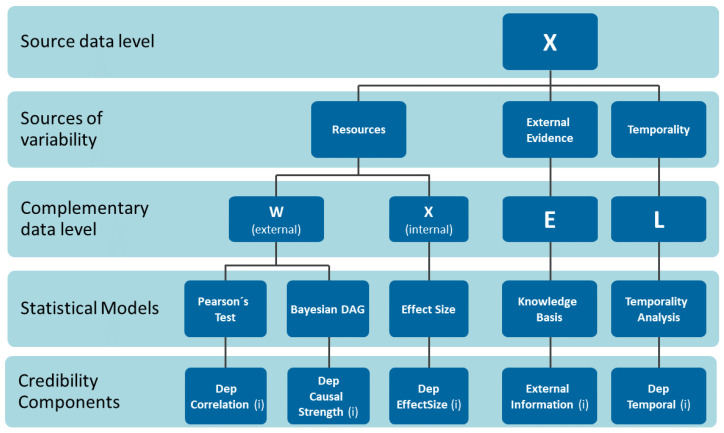
Schema of formation of the Credibility Index.

Figure 3 shows the different layers in the treatment of dependencies with which the credibility index is formed. Initially, the data features are incremented from external features (*W*) created from statistical models. The dependency by correlation is analyzed with the support of the statistical model Pearson Test and generates the DepCorrelation component variable. Causal dependence is more elaborate and uses the statistical model Bayesian inference producing the strength variable. Internal characteristics such as the size effect produced by the interactions between factors allow for the formation of the size effect component. The external evidence comes from the knowledge bases. Finally, the credibility index variable DepTemporal component is formed from a statistical model that elaborates on a temporality analysis at the complementary level of data L.

Consider an evidence generation framework in which a relationship structure in the data consists of a subset of covariates *X* that explain the phenomenon of interest *y*. Suppose further that a set of external factors *W* affects the phenomenon of interest *y* in the same way. Taking this frame of reference, the main elements that make up the proposed credibility index are explained below.

#### 4.2.1. Bayesian DAG

Under this scheme, the set of factors *W* represents those variables that can affect *y* but the effect of which we wish to isolate. One way to do this is first to identify whether the variable *y* is caused by *X* or by *W*. We then have to study the behavior of the conditional probabilities:

(1) P(y|X)
(2) P(y|W)
(2) P(y|X,W)

It would be the simplest interaction context between these sets of variables and can model more complex structures through a graph (DAG). The objective of this representation is to determine the causality of the variable *y* to rule out relationships in the base that may generate false evidence. This process constitutes the first pillar of the model. From it, it is possible to estimate the structure of the DAG under a Bayesian approach Markov Chain Monte Carlo (MCMC) and to determine which cases we could identify a dependence relationship because it comes from a non-causal source in the data.

One of the outputs within a Bayesian estimation process is the arc force. This variable allows for summarizing in percentage terms the frequency with which traversed each relationship (edge) in each simulation of the MCMC algorithm. *DepCausalStrenght(z)* is the variable that represents the strength of the arc in every relationship of the DAG for each element *z* in the population and is the first variable that is part of the proposed Credibility Index.

#### 4.2.2. Pearson’s Test (Correlation Analysis)

Although correlation and causality are not equivalent concepts, and although causality can play a more critical role in the background, both properties are highly desirable. Correlation describes behaviors in which the occurrence of one event is directly related to the occurrence of another. These aspects are essential in predictive modeling and automated learning issues since the performance of the models depends directly on this characteristic in the data.

Pearson’s test is a non-parametric statistical method used to test hypotheses in which it is desired to know if there is a significant difference between any two distributions. For this reason, it is helpful to determine when there is a substantial effect of an external variable. To that end, the distribution under the impact of this variable is studied and compared against a “placebo”. If the null hypothesis is rejected (equality in distribution), then evidence of a significant effect is obtained.

We are interested in observing relationships in which the variable *y* is caused and strongly correlated with *X* and not by *W*. The idea is to use Pearson’s test to determine the relationships that appear to be a strong effect on the set of covariates *W* and to adjust them. The use of the following test is then proposed:

- $H_{0}y=y|W$ : The *W* causes the *y*.
- $H_{1}y\neq y|W$ : The *W* does not cause the *y*

If the *p*-value obtained by the test is small, then it is rejected $H_{0}$ in favor of $H_{1}$. We are interested in preserving those cases in which the null hypothesis is not rejected. Since under $H_{0}$, there is no evidence of a correlation effect of *y* with *W*. Then, DepCorrelation(z) measures credibility at the registry level in terms of the correlation being the second component of our index.

**Figure 4:**
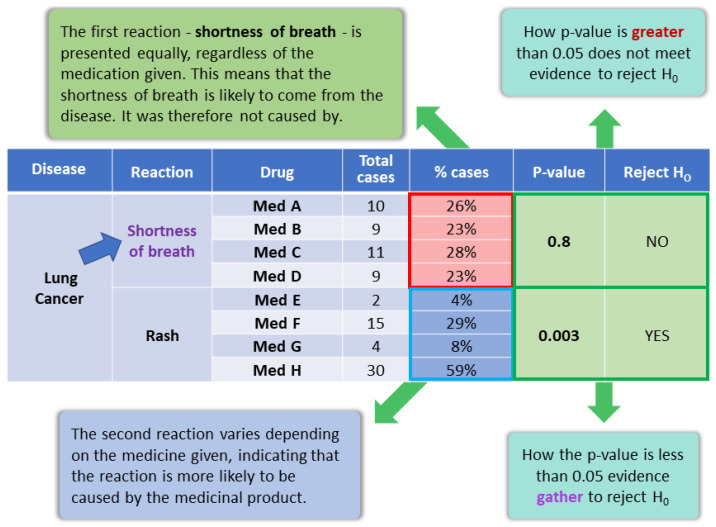
Pearson’s Test simulation for correlation analysis.

Figure 4 shows a hypothetical example of a Pearson test involving medications used by a patient and related symptoms and reactions. For a given symptom, the distribution frequencies are evaluated and a correlation is analyzed.

#### 4.2.3. Effect Size

There are often contexts in which the objective variable y can be explained through a series of factors, although it is unknown whether it was caused by one of them, by all of them, or by a combination of them. As the number of interactions that affect the same variable grows, the relationship becomes less evident, and the conclusions obtained through it lose quality.

To measure the degree of interactions in *X* on the target variable *y*, the design of this component is proposed that summarizes these interactions as follows. Suppose that *X${}_{i}$* is a categorical variable with c categories in *X* and $I_{j}$ is the variable indicator of category *j*th:

- $I_{j}\left(Z\right)=1$ if individual *z* belongs to category j
- $I_{j}\left(Z\right)=0$ if the individual *z* is not in categories

We then define the effect size for individual z as the incidence rate of z in each category:

$$Effect_{Size}\left(z\right)=\frac{1}{c}\sum_{j=1}^{c}I_{j}\left(z\right)$$

Then DepEffectSize(z) is a measure of interactions and is the third component of our index.

#### 4.2.4. Temporality Analysis

There is often the possibility to identify and gain insights from temporal trends in the data relationships. These trends can contain linear or non-linear patterns as time passes. These observations make it possible to obtain conclusions about the variable of interest from temporal trends associated with the set of covariates *X* that can lead to decisions about the target variable *y*.

These trends can be modeled through linear regression techniques to evaluate their level of statistical significance (α) and to be able to conclude them. Let

$$L=(L_{1},\ldots ,L_{k}),$$

be a set of covariates with complementary information to *X*, which contains temporal information about the study’s subset of observations. A simple linear regression analysis is proposed to determine the trend of each covariate $L_{j},\forall j$ associated with each individual through “*L*”. We then have a model of the following form:

$$L_{j}=\beta_{0}+\beta_{1}*t+\epsilon_{j};\;j=1,\ldots ,k$$

$$\epsilon_{j}\;Gaussian\left(0,\sigma^{2}\right);\;j=1,\ldots ,k$$

where *t* represents the time factor, $\beta_{1}$ is the slope associated with the regression, and $\beta_{0}$ is the ordinate to the origin. Under this linear regression model, $E_{j}$ represents the white noise associated with each estimate following a Gaussian distribution with zero mean and variance $\sigma^{2}$. This representation is the most common linear regression model, from which the following hypotheses are of interest:

$$H_{0}:\beta_{1}=0\;\mathrm{vs}.\;H_{1}:\beta_{1}\neq 0$$

This hypothesis test allows determining the statistical significance of the regression since $\beta_{1}\neq 0$ then time (*t*) affects the covariate $L_{j}$. More so, if $\beta_{1}>0$, then the effect is increasing and decreasing in the case $\beta_{1}<0$. It is then sought to reject the null hypothesis $H_{0}:$$\beta_{1}=0$ to have statistical significance in the trend.

Once a trend factor for each individual has been determined for the variables

$$L=(L_{1},\ldots ,L_{k}),$$

it is possible to validate whether an increase or decrease in the values of these variables can be associated with the target variable *y*. Formally, it is $\left(L,Y_{L}\right)$, the pair of evidence obtained from the temporal analysis. S defines the factor of timing for each z element within the set of covariates

$$L=(L_{1},\ldots ,L_{k}),$$

as follows:

- DepTemporal(z)=1 if the relation $\left(X_{z},y_{z}\right)$ is contained in $\left(L,Y_{L}\right)$
- 0 if the relation $\left(X_{z},y_{z}\right)$ is not contained in $\left(L,Y_{L}\right)$.

#### 4.2.5. PCA Method—Components Integration of Credibility Index

Figure 2 shows the flow for the Credibility Index formation process in a generic way. This approach can be applied to any domain. The CI is formed from four components: The first is related by relationships of dependence identified with a causal inference method. The second component arises from the correlation test between the entities under study. The third component comes from the interaction between elements that produce the effect size. The fourth member is formed by the dependence observed in a temporal phenomenon.

The index is composed through the linear combination of the data by the product of the first eigenvector of the matrix of the variances and covariances as *S = Var $\left(V^{t}\right)$*, where *$V^{t}=\left(DepCausalStrenght,DepCorrelation,DepEffectSize,DepTemporal\right)$* in such a way that the following equation gives the proposed credibility index in a general way:

$$\begin{array}{cc} IC_{PCA}= & e_{1}*DepCausalStrenght+e_{2}*DepCorrelation+\\ & e_{3}*DepEffectSize+e_{4}*DepTemporal \end{array}$$

where the vector $e=(e_{1},e_{2},e_{3},e_{4})$ corresponds to the first eigenvector of S.

Later in the next section, this stream gains the attributes of the ADR domain.

## 5. Experiment—Material and Methods—Model Applied at ADR Domain

### 5.1. Knowledge, Contextual, and Semi-Structured Information

An electronic medical record stores the patient’s clinical information and forms the primary data source from EHR. The data that comprise the EHR can be categorized into observational, contextual, and knowledge. Observational data are extracted from databases and electronic systems that build the EHR. The most important record is the clinical note. Medications can be indicated for the patient undergoing outpatient treatment, in the office, by issuing prescriptions. In case the patient is hospitalized, undergoing treatment, or receiving new dosages of medications, the frequency, volume, type of administration, and dosage are usually changed, characterizing contextual information and increased risk of ADR. Data obtained through access to EHR in databases are usually structured, but there are other types of representation.

The Clinical Notes are formed by unstructured data, where the health professional records, in free format, various information, characterizing a high dimensionality of information. Some data incorporate an aggregate business rule, known as semi-structured data. This situation occurs with laboratory tests and tests determined by domain knowledge information. For example, the normal reference values for the blood glucose parameter are between 70 and 99 mg/dL. When blood glucose is below 70 mg/dL, hypoglycemia may affect the patient. When blood glucose is above 99 mg/dL, the patient is in the pre-diabetes range, and the patient is considered diabetic if this measurement is greater than 120 mg/dL.

### 5.2. Sensors and Data Sources

**Figure 5:**
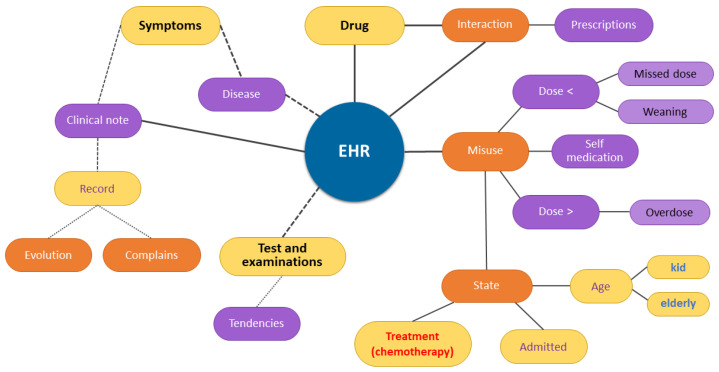
Mind Map—ADR from EHR.

Figure 5 demonstrates the mental map of the domain of ADR. Patient demographics are aggregated with diagnostic data such as the disease being treated and comorbidities. The main entities of the EHR model are the patient’s registration and demographic data relating to drugs, reactions, symptoms, diseases, tests, and prescriptions according to the patient’s condition (hospitalized or undergoing outpatient treatment). The collection of laboratory tests can determine the trends in blood parameters that may indicate ADR.

The heterogeneity and multimodality of data sources bring with them a high degree of uncertainty, including redundant and ambiguous sources of information. For example, duplicity—where the same data comes from two different sources presenting the same value—requires choosing a predominant source.

#### 5.2.1. Knowledge Bases

The structure for ADR analysis is formed from a domain model, which relates diseases, medications, and adverse reactions. The model uses two knowledge bases, the International Classification of Diseases *(ICD)* and an ontology of adverse effects [24], to search for known reactions that can be used as the Medicine leaflet package [24] contains knowledge-type information. The ADR record represents drug–reaction relationships for which there is already a clinical precedent, tested, recognized, and accepted, meaning-domain knowledge.

#### 5.2.2. ADR Base General Features and Volumes

The general characteristics of the database are described below.

(a) The database contains records that relate reactions and medications that have been administered to patients since 2019.
(b) The database consists of 81,740 such records and 11 columns describing, among other things, patient identification, date of birth, gender, clinical department, reaction, and administered medications.
(c) The records reported in the database correspond to 5937 patients treated in this period. The most crucial variables in the database are the medical area of origin, the drugs administered, and the symptoms or reactions complained about and observed. From these variables, the frame of discernment DST is built to estimate the belief of ADR from drugs applied or by the natural evolution of the disease.

**Figure 6:**
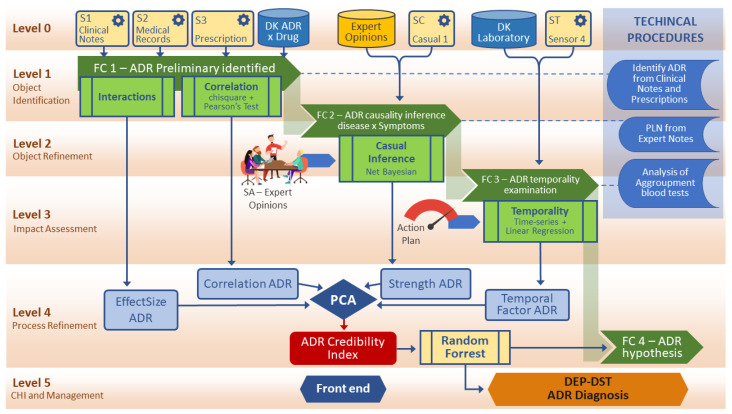
DEP-DST Credibal BPA applied to the ADR domain.

Figure 6 shows that the elements drugs and the entities under study are ADR or can be symptoms or reactions. The dependence relationship is weighted by the variables on correlation, causal inference, and temporal analysis. This figure includes the sensors and data sources detailed below:

- Clinical note—from a source of examination of the patient, an unstructured format that requires the use of NLP techniques for the extraction of medications and symptoms that may indicate ADR is presented. The clinical notes report an assessment of a patient’s condition by a health professional. They contain data on complaints described by the patient, the clinical and general status observed by the health professional, data recorded on the evolution of the disease, and the results of markers and tests;
- Prescription—from a source of outpatient prescriptions, it contains the dispensations of medicine for patients who are treated at home and outpatient clinic, who are not hospitalized;
- Medical Prescription—it contains the prescriptions of medicines for hospitalized patients. It is contextual information; usually, drug applications occur intravenously at more potent doses and demands, increasing the incidence of ADR;
- Chemotherapy—from the sensor that gathers data on patients undergo chemotherapy;
- Pathological Tests—with varied frequency, data are obtained from the sensors of laboratory tests, with the most common being blood and urine tests. They report structured information on the reference values of the result of the markers. The use of medical knowledge-type information is required to assess deviations in behavior and detect trends.

### 5.3. MSIF—Data Fusion Process

The following analyzes the information obtained and the processes carried out at each level of the proposed MSIF. More details of the model are in Section 3.1.

#### 5.3.1. Level 0—Signal and Pre-Processing

This level represents the data request and involves pre-processing for sensor refinement. The emphasis is on acquiring data from different sources and sensors in distinct formats. It is necessary to configure specific parameters to capture data. Periodicity, temporality adjustments, address, form of access of sensors, and sources are calibrated. The sensors that contribute to this fusion process gather the basic information of patient registration, evolution from clinical notes, outpatient prescriptions, hospitalization, and chemotherapy treatment. There is also a sensor that captures laboratory tests. The data are retrieved hourly, twice a day, or once a day in night processing. After the data capture is configured from the sensors, it performs the analysis, evaluation, and prediction of the observable states of this information. This assessment enables the removal of noise and redundancy in allocations and measurements, optimizing the processing of the following levels. This configuration meets the conditions for pre-processing and prepares the environment for the next level of MSIF.

#### 5.3.2. Level 1—Object Refinement

Level 1 emphasizes the association between data, making it possible to identify and locate ADR from properties and attributes. The combination of data occurs to obtain the most reliable and accurate estimate of ADR. Space and timing references are aligned, acquiring ADR objects’ interest through the domain’s multiple sensors. The sources obtained at the zero level are associated, requiring homogenization processes to enable the correct association between the patient’s medical records, and the registration information present in the EHR its related. This level provides the first process fusion center, providing the essential infrastructure for ADR detection from the newly merged information. The ADR domain knowledge describes the possible ADR arising from medication intake and interactions. The model can obtain the information provided through search procedures, including NLP-specific techniques from the information infrastructure associated at this level. The focus is on identifying ADR. From the high-dimensional unstructured texts that occur in the description of a clinical note, the elements that constitute an ADR are extracted: medicines and reactions. Combining possible ADR reactions from intake medications and interactions captured are checked against the knowledge base. The interactions between drugs applied to a patient are performed by implementing the EffetcSize variable that integrates the CI. At level 1, the first dependence analysis of the model occurs through correlation analysis.

From the drugs and symptoms assigned to the patient, it is possible to perform a correlation analysis. A Pearson’s test is applied, which allows for correlation analysis of this possible dependency relationship. Thus, the DepCorrelation variable is produced, where a potential false ADR receives a lower weight. Figure 4 shows a simulated example of a Pearson test involving medications used by a patient and related symptoms and reactions. The distribution frequencies are evaluated for a given symptom, and a correlation is analyzed, as defined in Section 4.2.2.

#### 5.3.3. Level 2—Refinement of the Situation

This level involves the evaluation of ADR identified preliminarily. At this point, Ref. [20] used situational awareness. The prescriptions and chemotherapy sensors typically occur in cycles, in which the same drugs are applied for several consecutive sessions. They are applied via injection, dissolved in solvents and auxiliary drugs that are generally not in the ADR influence zone. The data need to be consolidated, with the cleanup of redundant data. Thus, the granularity of the ADR candidate registers involving the combination of drugs and adverse reactions extracted from clinical notes in the previous step is adequate. From the fusion center, the dataset is increased with these adjustments and experts discuss the preliminary results. The Bayesian network results are about thet ADR. Clinical notes are generated in special formats, and new extraction procedures are applied using NLP and knowledge bases. This is a crucial step for the other levels of MSIF, relating contextual information acquired on ADR. The ADR at this point is a potential record, which needs to be evaluated and go through the process of estimating the following stages. At level 2, the second dependence analysis of the model occurs through bayesian inference.

The natural evolution of the disease itself may be the actual cause of a previously supposed adverse reaction. In reality, it would be a symptom, a sign of the disease.

**Figure 7:**
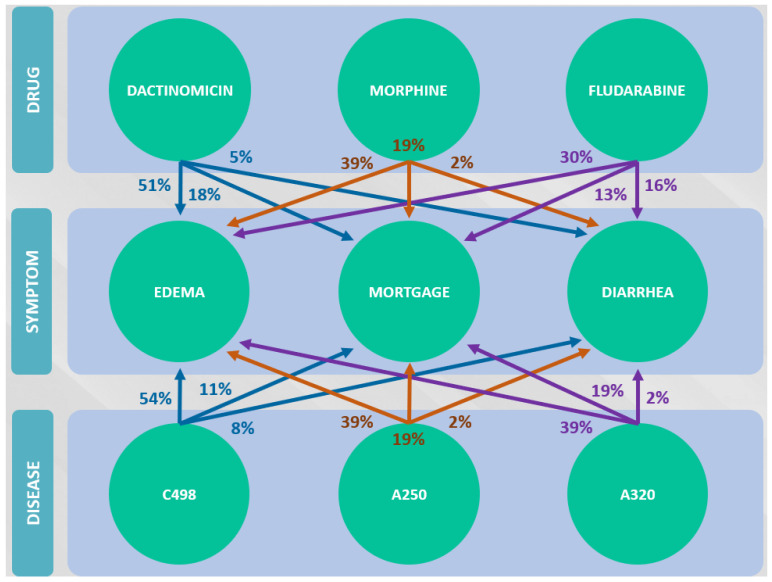
Bayesian DAG.

Table 1 and Table 2 demonstrate the conditional probability matrices obtained from the Bayesian network. Table 2 mentions the conditional probabilities obtained by the Bayesian network that address the symptoms of the diseases. Table 1 addresses ADR relative to drugs. The disease can be the factor that leads to symptoms that can be mistaken for drug reactions. This dependency relationship has to be evaluated to obtain more accurate results. Figure 7 illustrates the Bayesian DAG. To more accurately measure the probability of presenting a symptom or ADR when taking a drug, from the analysis, it is necessary to identify the effect that the disease causes on the symptom and not corresponding to ADR.

Note that, in Table 2, disease c498 has a conditional probability of 54% for the edema symptom, which in turn maintains a conditional probability of 51% being ADR related to the drug Dactomicin.

#### 5.3.4. Level 3—Impact Assessment

The complete set of drugs and records candidates for ADR is available at this stage and allows for the refinement of the model in this third stage of fusion. The a priori knowledge established in the previous levels is interpreted to predict the estimates and effects. Then, the refinement of the ADR threat analysis is carried out, modeling the current situation to allow for future inferences.

Specialists perform an action plan where some markers are specified in pathological examinations, enabling the evaluation of threats. Sensors capture these markers such as glucose, urea, creatinine, hemocytes, and leukocytes. Based on the evidence from the results for these markers over a period of time, the occurrence of a tendency for a marker is verified, whether rising or falling. This trend may be related to ADR triggered by a given drug. For example, if the patient experiences a tendency to increase urea in the last tests, maybe some medication caused this variation. This fusion center encompasses the formation of a longitudinal dataset that allows for the subsequent temporality assessment seen. In some cases, a relationship of time dependence can be established, as seen in Section 4.2.4.

##### Model for Analysis of Pathological Examinations

**Figure 8:**
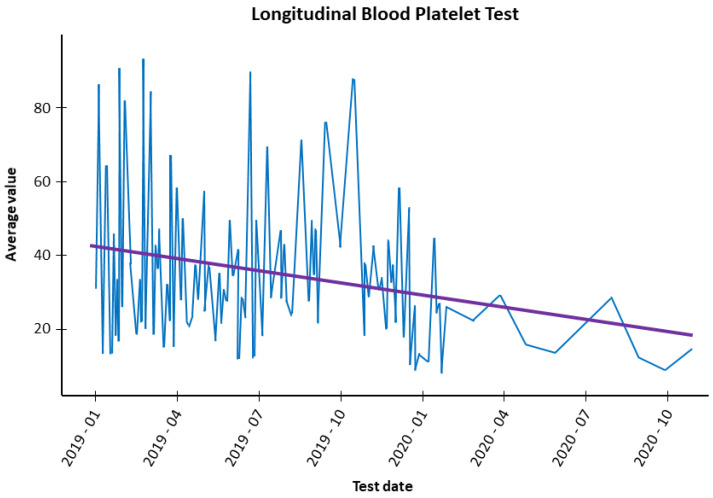
Linear Regression—temporal analysis of the impact of the drug under examination.

The methodology used is based on linear regression models and hypothesis tests, using the F-Test. Figure 8 shows the linear regression graph. The proposed model considers the information obtained from the patient’s blood tests, according to the following:

(a) The database used in this step consists of 2338 medical records and 11 variables.
(b) The database contains the results corresponding to creatinine, glucose, urea, and total blood count values.
(c) Seventy-eight patients were considered on this basis.

At level 3, the third dependence analysis of the model occurs through temporal analysis.

Figure 8 demonstrates the linear regression graph and temporal analysis of the impact of the drug under examination.

As a particular example, consider the case of the patient who has been administered with Morphine. The platelet level of this patient was counted over time from January 2019 to October 2020.

1. A linear regression model was adjusted to determine the trend of the monitored value during the application of the drug over 360 days. In this case, a decreasing trend can be observed in the platelet count value over time. According to medical experience, this decrease can cause the symptoms: bleeding, edema, asthenia, or neuropathy.
2. To test the effect of the drug on the value of the marker, a hypothesis test was applied:
  *$H_{0}$:* The drug does not affect the blood marker;
  *$H_{1}$:* The drug affects the blood marker.
3. For each blood test, the probability of presenting variations up or down as an effect of applying a drug is determined.

The statistical significance of the previous trend has been validated by a linear regression analysis of the time series of the patient. The results show evidence of rejecting the null hypothesis $H_{0}\beta_{1}$ = 0 in favor of $H_{1}\beta_{1}\neq 0$ when obtaining a *p*-value less than a significance of α = 0.05. From this, it is concluded that the drug affected the marker platelet. A decreasing trend effectively occurs in the value of the platelet study.

#### 5.3.5. Level 4—Process Refinement—Calculates the Probability of ADR and Applies CI

At Level 4, the goal of refinement processes is to close the model cycle. A model of evidence generation to ADR is implemented, characterizing the incidence of this relationship between a drug and a given reaction or symptom. This level enables potential sources of enhanced information, such as trackers, new medications, new ADR, and sensor allocation. The parameters used at different levels, such as granularity, capture frequency, and news sources, are optimized and adjusted. An effective technique is to include pre-processed data sources from an interface. A more accurate causality analysis can be inserted here, adjusting parameters for inference with Bayesian networks to improve the model.

The previous levels have their performances monitored. There is a closing of targets at this point. The identification and estimation of ADR serve as a basis for forming the CI. The CI guides the construction of the evidence generation to construct the DST frame of discernment. We detail the process of credibility index generation applied to ADR, the DST evidence generation process, and the application of a benchmark.

##### Credibility Index—Implementation

Modeling an ADR context is challenging if the symptom may come from the disease and would not be an ADR caused by the use of medications or it may be the result of an interaction of two or more drugs. This study establishes the criteria we reviewed throughout the research to assign measures to the credibility of an ADR. The functions used to quantify these parameters are formally detailed.

1. DepCausalStrenght: the value referring to the strength of the arc from the inference of the Bayesian network:
  $$DepCausalStrenght_{A}DR\left(z\right)=stregnht\left(z\right)$$
  As described in Section 4.2.1 and seen in Table 1 and Table 2, the conditional probabilities are calculated, generating the variable strength and the graph that allows for Bayesian inference such as Figure 7.
2. DepCorrelation: The complementary value of *p*-value obtained with Pearson’s test was used to quantify the effect of the disease on ADR. For each patient, a value of (1 − *p*-value) was calculated to measure the correlation component of ADR credibility. To this end, Pearson’s chi-square test was used, modeling the hypotheses:
  - $H_{0}$: The disease causes the reaction or symptom.
  - $H_{1}$: The disease does not cause the reaction/symptom
3. DepEffectSize: The size of the effect has been measured to consider that an ADR in which several drugs interacted is less believable than one in which the effect is isolated. If n is the number of medications that patient *i* took, then
  $$Effect_{Size}\left(z\right)=\frac{1}{c}\sum_{j=1}^{c}L_{j}\left(z\right)$$
4. DepTemporal: If the ADR potential is contained in the TemporalityBasis, the record is marked with the boolean value one. Otherwise, it is zero. This function is essentially a boolean variable indicator.

We implement a PCA method to represent the variables in a smaller dimension. This technique summarized the three belief factors (size effect, knowledge effect, and causal effect) in a single indicator. The method defines a weighted linear combination of belief factors by assigning different weights to each of them.

The method seeks the direction of maximum variability in the data to summarize the most significant amount of information available in the first components (new dimensions). Thus, PCA first dimension can be used as a linear indicator that summarizes the behavior of belief factors.

##### Evidences Generation—Random Forest

We use Random Forest to evidence generation technique. A Random Forest is a Machine Learning model that fits a series of decision trees into subsamples of the data set and uses averages to improve prediction metrics. ADR involves a high-dimensional context. In this type of scenario, it is essential to control the complexity and size of the trees. A way to control this is to set the values of predictive accuracy and control overfitting. The variables are always randomly permuted in each division. Therefore, the best division found may vary, even with the same training data.

##### Benchmark Method

The information obtained by analyzing time series from time evidence obtained in the domain is aggregated, indicating the dependency relationships between variables. Finally, there is a block referring to causal inference that uses a Bayesian network, where the strength of the arcs is an indicator of weight for the formation of the CI. In each block, a BPA (hypothesis formation) is formed that corresponds to mass functions.

Note that there is a conjecture of hypotheses corresponding to the BPA created from each level onwards and evolves as the stages of the process pass. They are interaction factors that combine and produce BPA with a mass value m for each hypothesis.

**Figure 9:**
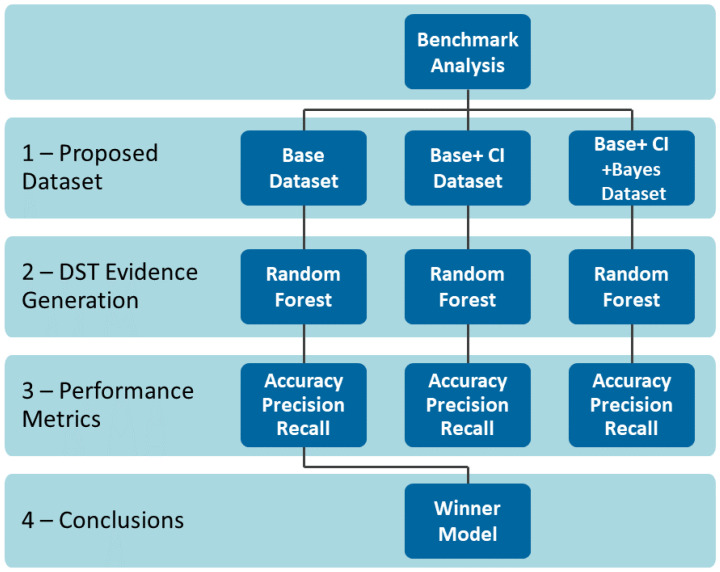
Benchmark method.

Figure 9 demonstrates the benchmark method.

- Evidence Generation DST: Evidence is generated for a DST context through a learning model Random Forest trained in the three different dataset scenarios.
- Proposed Models: the first scenario includes a dataset base with frequentist probability. The second scenario contains a dataset incorporating the PCA credibility index without the Bayesian variable. A third scenario that also considers the variables from inference Bayesian.
- Performance Metrics: three performance metrics were used to quantify different aspects and have an objective overview of the performance of the models, accuracy, precision, and recall.

#### 5.3.6. Level 5—Process Management and Computer–Human Interface (CHI)

Finally, Level 5 allows for the management of the model and the human–computer interaction (HCI), for the selection of medications and adverse reactions, and for the specification of visual methods of presenting the results.

**Figure 10:**
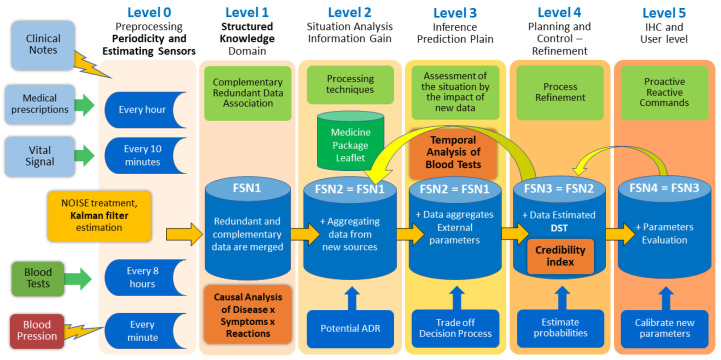
Information fusion big picture.

Figure 10 demonstrates the MSIF levels, highlighting the gain in information at each stage and showing the general vision of the process of information fusion. The ADR candidate record appears at level 1 when EHR’s information about drugs, reactions, and symptoms are accessed. At level 2, domain knowledge is used to validate reactions and medications and to perform the causal analysis to isolate effects caused by diseases and not by drug consumption. At level 3, the highlight is regression analysis in blood tests, seeking temporal trends of changes caused by drug intake. Level 4 presents the practical application of the CI, which allows for the estimation of probabilities on a more reliable dataset. The information flows between the levels with continuous feedback that enables the evolution of the MSIF. Finally, at level 5, model parameters are calibrated, usable, and interface oriented.

In this last level of MSIF, the resources of interaction between the human being, the system (HCI) are determined, and management aspects are reviewed. Cognitive refinement is performed, and initiatives related to process management take place. Visual methods are specified to display the forms and output information of the fusion process results. The information is used as knowledge for specialists working on selecting medications and related reactions that are the focus of attention for problem management.

### 5.4. Explaining How the Method Works

The model starts with data from an information fusion process gathering patient data, clinical notes, medical prescriptions, and prescriptions obtained from inpatients. In the medical prescriptions, the medicines applied to the patient are registered. The ICD, the international disease code, informs about the pathology affecting the patient. Patients are exposed to different medications, remembering that in case of comorbidities (e.g., diabetes and high blood pressure), the amount of medications increases, generating drug interactions. During a clinical consultation, the patient exposes some complaints to the doctor. The doctor, in turn, observes symptoms that the clinical examination allows us to assess. These complaints and observations are registered in a clinical note (NC). There is even an additional source of data, necessary for some cases, in which the patient’s laboratory tests.

A patient undergoing cancer treatment undergoes a series of tests over time, at least one exam a month. If they go through periods of hospitalization, this frequency of exams is more intense. The collection of laboratory test data allows for the generation of a time series. In some cases, trends in the variation of some blood parameters can be observed (such as glucose, urea, creatine, and leukocytes, among others). Some of these variations in laboratory parameters may indicate ADR. Several symptoms and complaints may be ADR or may arise from a natural evolution of the disease being treated. The application of the model in the ADR domain determines the relationships of dependencies between different sources (recipes, prescriptions, clinical evaluations, and patients’ complaints) and confronts them.

The proposal to create the CI allows for evaluating causal and temporal dependencies by causal inference the relationship between disease, symptoms, reactions and drugs,reported in the variable strength obtained by the Bayesian network inference.

The association between medications and reactions/symptoms is mapped by the variable DepCorrelation, which relates drugs and symptoms through a multivariate analysis of a Pearson chi-square test, evaluating the correlation and, in the case of an outlier in the distribution, the proportion presented in a hypothesis test (*p*-value).

Finally, the CI is formed by a temporal component, extracted from a linear regression obtained by the time series of exams, when these allow for the formation of high or low trends in blood parameters, leading, in some cases, to possible ADR intensified by the use of medication over time. This function is performed by the DepTemporal variable that takes care of temporal dependence integrates the CI.

Take a scenario with a diagnostic decision in an environment of uncertainty. The model allows for the generation of evidence with a random forest algorithm. Distinct dataset scenarios alternating the components of CI were used, allowing for the evaluation of the evolutionary effectiveness that the application of these variables to be improved in the model.

### 5.5. Final Considerations about the Proposed Model

This model was implemented at a cancer hospital and could be applied in other health institutions. Transfer Learning can be an exciting opportunity, especially in the application of the proposed model in other health institutions interested in an instance in their environment. Two studies relevant to applying transfer learning are [25,26]. They used a learning technique that can significantly optimize training time and the need for notification and case labeling activities.

## 6. Results

In this section, the results are presented. A benchmark analysis was performed with evolutionary versions of the credibility index. To observe the proposed DEP-DST method, the metrics obtained in this study were compared with the literature. To assess the ability to generalize the results, an externally based test was carried out in another domain other than ADR. The section will begin with the explanation of the CI distribution to continue with the approach of comparative studies.

### 6.1. CI’s—Contribution to the Study of Independence and Variability

The distribution of the CI in a histogram demonstrates the high value of this variable. When the CI has a high value (80% of the time), the RF accuracy improves. When it has a low value, it also improves accuracy, in the sense of avoiding false positives, as it does not recognize it as ADR. It was decided on a benchmark analysis to demonstrate the value added by the CI. This analysis was carried out in three stages, with CI component variables being gradually added to the dataset scenarios for the comparative table.

**Figure 11:**
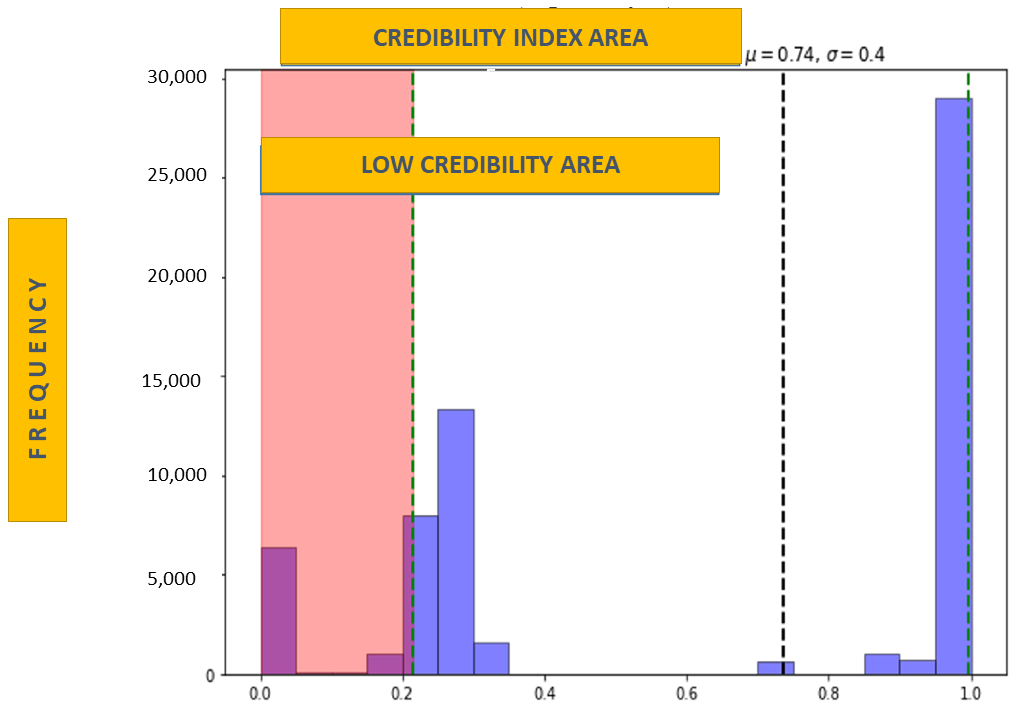
Credibility Index Distribution.

Regarding the results obtained for the credibility index, Figure 11 shows how the CI was standardized to have a percentage scale index between 0 and 1. The cut-off point for the index with the upper limit of the low credibility zone is now considered the first interquartile value in which the interval (0, 0.21) is obtained.

Regarding independence and variability during evidence generation, the model allows for the combination of different sources in the DST discernment framework. The first source would be the BPA obtained by the RF method that used the CI and started from an EHR dataset from the DST-DEP data fusion process. A second source independent of the first one can be obtained from a knowledge base such as the medicine leaflet package. Additionally, unbiased and independent expert opinions can be added.

Regarding the issue of variability, the improvement would be on the side of precisely controlling external variables such as the disease. From the use of the Arch strength variables and Pearson’s correlation with the disease presented by the patient, obtained from the knowledge base of the (ICD), there is a better control of variability induced by external sources.

**Figure 12:**
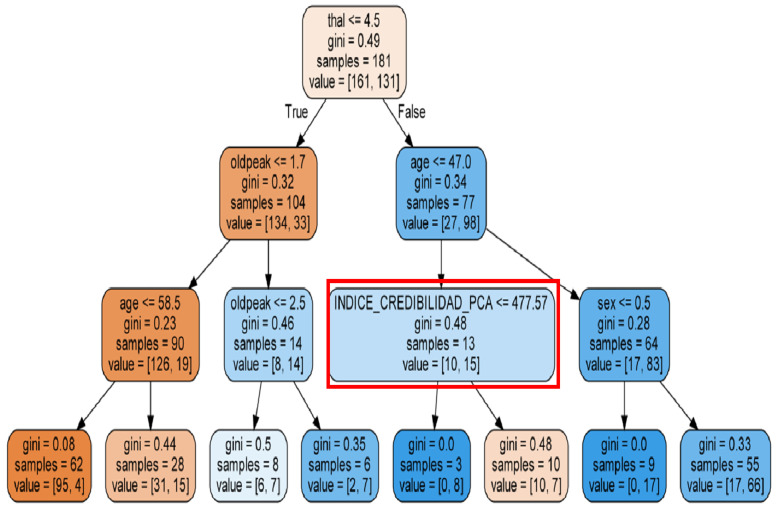
RF graph generating BPA, reflecting the importance of CI.

Figure 12 shows the graph that corresponds to the evidence generation process (BPA) followed by the Random Forest applied to dataset heart diseases, in which it can be seen how the CI (red box) is part of the model contributing to the decision-making process of one of the trees in the RF.

### 6.2. Results of Benchmark Analysis Confusion Matrices and Comparisons with Literature and External Base

**Figure 13:**
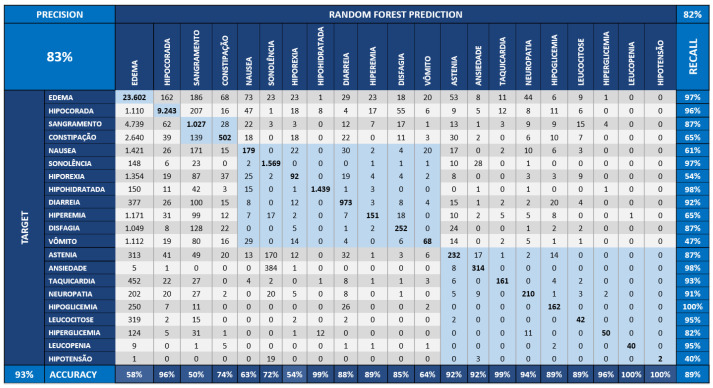
Benchmark 1—Base Model.

**Figure 14:**
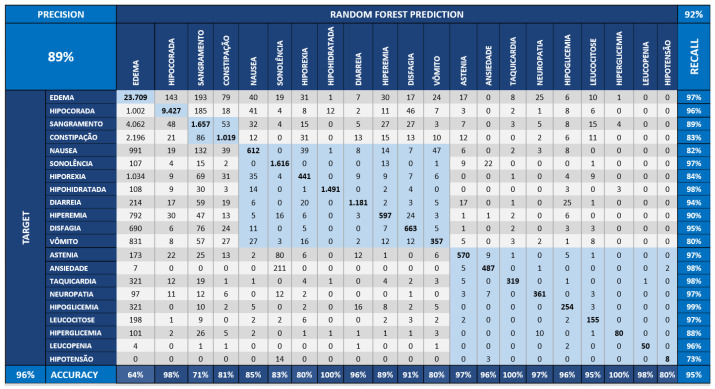
Benchmark 2—Base Model + CI.

**Figure 15:**
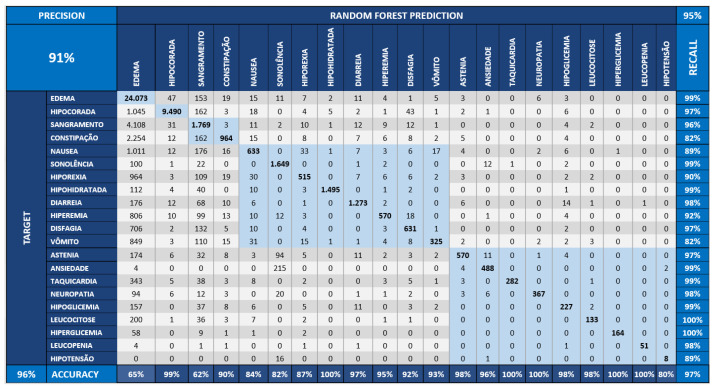
Benchmark 3—Base Model + CI + Bayesian Net.

As defined in the benchmark process, we performed the Random Forest method at three distinct dataset scenarios: the first, represented in Figure 13, displays the original dataset, named “base”. The second, the dataset is incremented with the CI, illustrated in Figure 14, and is formed by the effect size, temporal, and correlation effect components. Finally, the third scenario, described in Figure 15, shows the Bayesian variable strength added.

Figure 13 shows the confusion matrix obtained by executing the Random Forest applied over the dataset without the CI components. Figure 14 demonstrates the confusion matrix obtained from the Random Forest applied to the base dataset augmented with the CI components of association and temporality. Figure 15 starts from the dataset with CI and adds the Bayesian inference indicators from the strength component.

The values of the diagonal of the matrix of the Figure 15 are greater than a matrix of the Figure 14; this means greater assertiveness. The “TARGET” symptoms are the real ones (vertical) and those of the “RANDOM FOREST” are those learned by the model (horizontal). This behavior leads to greater assertiveness, which can be seen when accuracy increases from 81% to 95% in the second case (base model + CI + Bayesian component).

After executing the internal scenarios of the internal dataset, we executed the model on Cleveland’s ICU-heart base, which is widely used in diagnostic research for heart disease applying machine learning. We used [27], which obtained an accuracy of 85% in the best results from several studies that achieved an average of 80% accuracy. The model DEP-DST was slightly lower, a precision of 78.4%, accuracy of 76%, and a recall of 0,77%, achieving an F1-measure of 78%. The behavior of the DEP-DST in a new dataset achieved 90% of the best result and 95% average from several studies in [27]. The training was fast, and we believe that the adjustments of future work will be able to add new comparative tests. It is believed that we can reach the same result and probably surpass this comparative study, which should improve if specialists in the cardiology domain help improve our causal inference engine, which in the case of ADR, was the most significant variable. Probably if we use this causal inference at the base of the heart, we will achieve better results.

Table 3 consolidates the benchmark results between the dataset versions incremented by the CI components and finally augmented with the Bayesian variable strength.

Studies were selected from the literature that developed methods based on different techniques for the comparative analysis. Table 4 shows that the performance of the DEP-DST model proved to be superior to the SVM, DT, and deep learning models.

### 6.3. Explaining How Does the Method Generate BPA by the Random Forest Method

A Random Forest model is trained from the dataset. In the case of ADR, the model considers the patient’s current medication context and predicts the most likely reactions. The model was enriched with the individual belief factors that generated the CI during the training phase to improve performance. The general characteristics of patients are considered covariates and are used during the training phase to estimate the probability of having ADR. The BPA is constructed using the Random Forest method from covariates, IC-PCA, and individual belief variables (Depcausalstrength, Depcorrelational, Depeffectsize, and Deptemporal). After that, the generated BPA can be combined with the external information that contains the medicine leaflet package, using the Dempster rule.

### 6.4. Explaining How Does the Model Combine Sources to Generates Believes

Since the CI is a measure of the confidence of the ADR, its complement (1 − CI) would represent the uncertainty as distrust of the evidence in a closed world context. The model leverages the information provided by the Credibility Index to generate evidence based only on the most plausible data. This first BPA is generated with Random Forest and then combined with evidence based on external sources. Evidence from external sources may have a certain level of credibility for the experimenter. This level of credibility is also taken into account when calculating the final evidence with the Dempster Rule.

An example of running BPA to calculate the BPA corresponds to the “ADR PROBABILITY” field in the DASH and is the probability estimated directly by the RF. In the following example, it is inside the red box and corresponds to the value of 97.22%.

**Figure 16:**
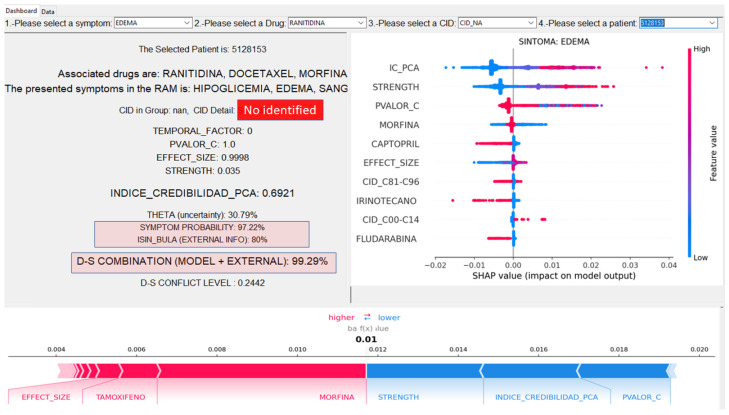
BPA explanation.

Figure 16 displays the functionality of the ADR front end. The user chooses the drug, symptom, disease, and patient. The model displays the probability of ADR, the variables that make up the CI calculation. The CI and uncertainty are displayed, along with the belief obtained from knowledge bases, in the case of the medicine leaflet package.

The patient consumed the drug Ranitidine and presented edema. Note that the system sought, in addition to Ranitidine, two other drugs—Morphine and Docetaxel—that were prescribed to this patient. The probability of Morphine generating edema is 92.7%. This is the BPA calculated by the system, based on the CI and its component variables. There is external information obtained from the medicine leaflet package that can be combined. Figure 16 shows the model performing the Dempster combination of BPA and external information, and the model attributes 99.5% belief to ADR.

## 7. Discussion

The three dependence treatment processes can be verified in Section 5.3.2, Section 5.3.3 and Section 5.3.4.

The experiment featured 5937 patients who were treated for 85 different diseases: 55% were women and 45% were men, with 45 years old being the average age, 10% being children, and 85% being older (>65 years old).

Initially, 63,000 clinical notes were recovered, which were recorded throughout 2019. From these clinical notes, approximately 8.7% mentioned drugs or symptoms, with 55,000 records selected at this stage. Over 10% of these records were generated after homogenization to generate medication and symptom pairs, generating 5600 records of possible candidates for ADR. Then, the clinical notes and prescriptions applied to the patient were combined, regardless of being outpatients, being used during treatment, or hospitalizations, which generated more than 6300 prescriptions combined with the candidate records for ADR from the developments recorded in the clinical note.

The pathology collaborated for 40,414 records generated by 911 of the 960 patients selected in the previous step. In other words, 95% of patients underwent some examination and with a reasonable frequency, with an average of more than four annual examinations per patient. It is known that patients hospitalized or in critical stages of treatment are tested more frequently. A procedure was then carried out to assess trends in the exam parameters, generating 4415 records of an upward trend and 3333 of a downward trend.

### SHAP Analysis

This topic is intended to present explanatory results of the SHAP technique. The following figures show visual explanations for predicting ADR using the *SHAP* technique [32]. Thus, in the red part of the graph, we have the characteristics that most influence the prediction of ADR and, in the blue region, the features that least influence the diagnostic of ADR. Taking the mean of the absolute value of the Shapley values, it is possible to estimate the global contribution of each variable to the model. After the CI, it is clear that the Arc strength assessed using the Bayesian DAG is the one that most contributes to the model, followed by the *p*-value of the correlation test and the effect size.

**Figure 17:**
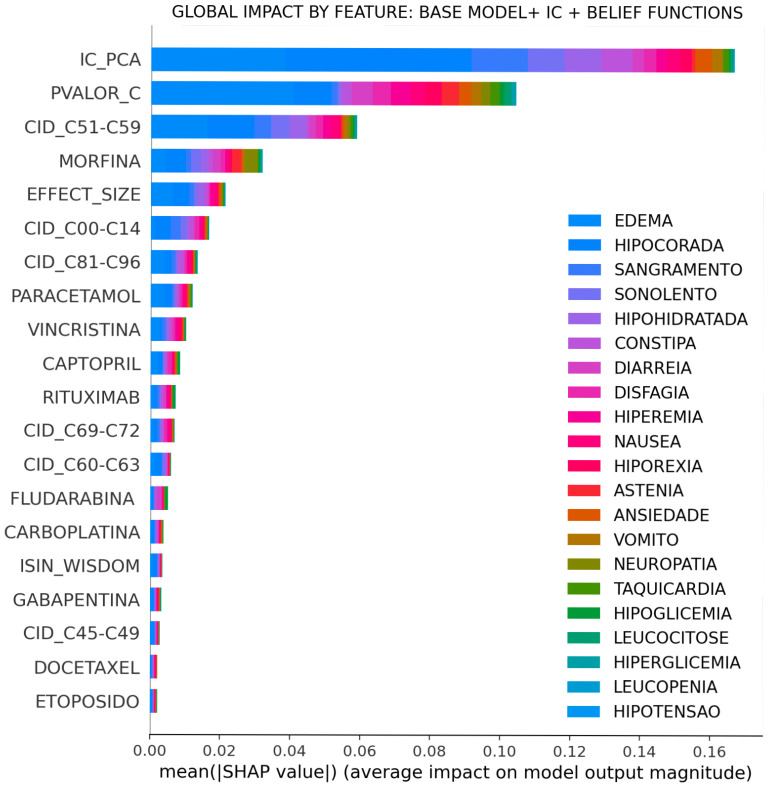
Shapley global impact for feature.

Figure 17 suggests that the CI and its components act as a first filter in the decision process, evaluating the level of belief in each record. Next, we have the variables associated with the effect of each drug, which end up adjusting the probability in the decision process. It is essential to note the fluctuation of drugs and diseases (represented by ICD) as scenarios vary.

**Figure 18:**
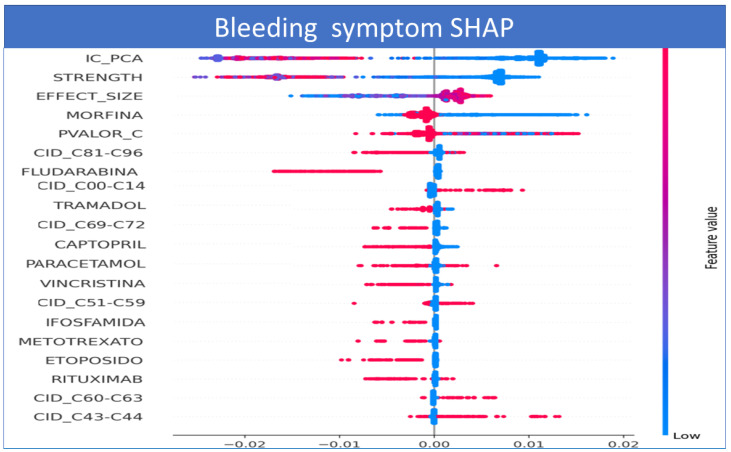
SHAP value impact—bleeding.

Considering the Shapley values for each output in Figure 18, it is possible to estimate the contribution of each variable to the symptom level. In this example, observe that higher values of CI contribute positively to the probability of the BLEEDING symptom, while lower values decrease the probability of the symptom. We analyze some drugs and observe that some medicines such as Docetaxel, Paclitaxel, and Irinotecan are positively correlated by SHAP. These drugs present a higher probability of ADR bleeding. This fact may indicate that the effect of these medications may affect the presence of this symptom or ADR.

**Figure 19:**
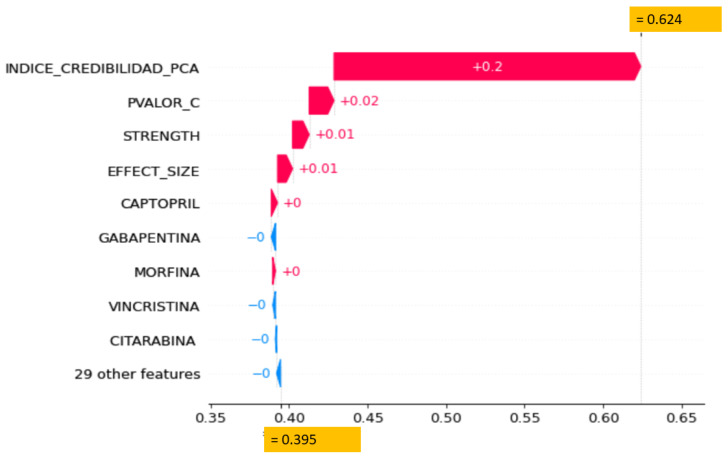
SHAP patient perspective.

Figure 19 presents the patient level, and SHAP offers a visualization that allows for identifying the contributions of each variable in the probability attributed to each observation (patient). For example, in this case, the model starts from the expected value of the symptom nausea (39.5%). Observe that this probability decreases or increases according to the effect of some drugs such as Citharabine, Vincristine, Morphine, Gabapentine, and Captopril. Also reflects a more significant impact in this case through the individual belief variables (DepEffectSize, Strength and DepCorrelation) and the IC-PCA index, ending up increasing the probability of the symptom up to 62.4%.

**Figure 20:**
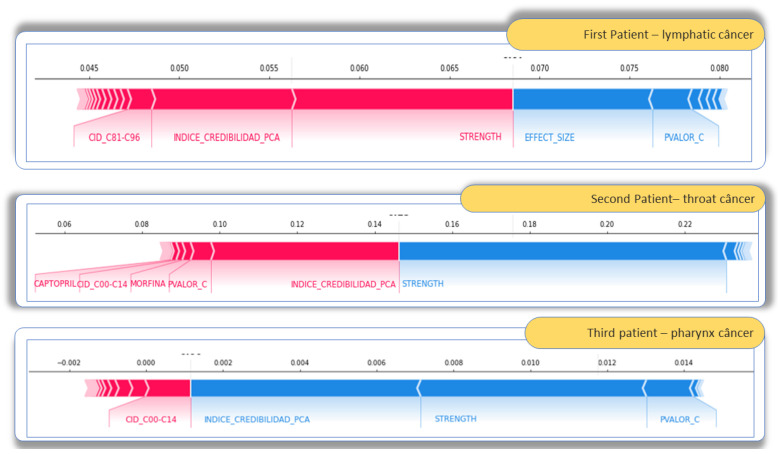
SHAP patient perspective summary.

Finally, the decision process in Figure 20 demonstrates that the SHAP model was chosen to explain several aspects of this decision-making environment:

(1) Each patient is a case because they have their disease and their immunological nature, and they take specific medications and comorbidities, leading to reactions and symptoms;
(2) It is possible to have a *SHAP* analysis per symptom/ADR; and
(3) Temporality contributes in about 10% of cases, and when it occurs, it is decisive.

## 8. Conclusions

An MSIF model was created using DST with reliability acquisition. The CI with the introduction of causation and time factors improves the hypothesis generation on the frame of discernment DST. The proposed model was applied in the ADR domain. Even in the timely evolution of future studies, the causal analysis demonstrates that several complaints of patients obtained in the clinical notes are derived from the evolution of the disease and are not an effect of drug intake.

As well as the analysis of time, data through linear regression proved to be efficient in detecting trends in the variation of blood parameters obtained from tests performed with patients. These time trends demonstrate a potential effect caused by using a given medication over time. The preliminary findings show that the presentation of the results obtained using DST proved effective for managing users.

The proposed CI reduces uncertainty, as it allows for a cut or categorization of records with low credibility, in the case applied, to be an ADR.

The disease is Factor C → B in the domain in which the result of interest is A → B, drugs that provoke ADR.

The benchmarking results show that it is possible to improve the performance metrics by incorporating the credibility index and the Bayesian DAG. The table shows an average improvement in the metrics used to measure the performance of 21% in the model using the Credibility Index and up to 25% when also considering the Bayesian DAG.

This scenario ensures that the disease’s usual symptoms can be confused with ADR. It would be a false positive to ADR. These signals are natural symptoms that should not be confused with reactions caused by medications. The study of dependency relationships, by causal inference and correlation, proved to be relevant to increasing the model’s precision.

## 9. Future Work

There are three initiatives for future work. Initially, the model applied to ADR needs to contemplate comorbidities, secondary diseases that can cause ADR. The PCA model can be replaced by the Kernel PCA version [33]. The basic idea of the PCA kernel is to use a non-linear kernel function (k) instead of the default scalar product. We implicitly run PCA on a high-dimensional F space that is not linearly related to the input space. This space change allows for obtaining linear spaces where the traditional PCA benefited, increasing the possibility of capturing a more significant variance in the first components and consequently obtaining a better rank when using the first eigenvector of the components. The efforts made until this work was conducted included the construction of the BPA, generating evidence for a better possible diagnosis. It will create significant future results on unpublished ADR and highlight the uncertainty for better decision-making. A process of generating ADR alerts can be established, which is, therefore, the merged base of the application used to support an ADR risk monitoring activity. Another opportunity will be the use of transfer learning techniques, mainly to use new model instances in other public health institutions interested in this research topic.

## Figures and Tables

**Table 1:** DAG probabilities for drug–reaction.

DRUG	Reaction No	Reaction Yes	Reaction
Dactomicin	49%	51%	Edema
Dactomicin	82%	18%	Mortgage
Dactomicin	95%	5%	Diarrhea
Morphine	81%	19%	Mortgage
Morphine	61%	39%	Edema
Morphine	98%	2%	Diarrhea
Fludarabine	84%	16%	Mortgage
Fludarabine	70%	30%	Edema
Fludarabine	87%	13%	Diarrhea

**Table 2:** DAG probabilities for disease–symptom.

DISEASE	Symptom No	Symptom Yes	Symptom
c498	46%	54%	Edema
c498	89%	11%	Mortgage
c498	92%	8%	Diarrhea
A250	61%	39%	Edema
A250	81%	19%	Mortgage
A250	98%	2%	Diarrhea
A320	61%	39%	Edema
A320	81%	19%	Mortgage
A320	98%	2%	Diarrhea

**Table 3:** Benchmark analysis (performance).

Dataset	Description	Accuracy	Precision	Recall	Improve
ADR Base Dataset	Includes the variables generated under the context of multiple interactions and binary variables that indicate the presence or absence of the drug in the period of observation of symptom	81%	59%	61%	’nA’
ADR Base Dataset + CI	Includes the variables of the Base dataset plus the percentage of credibility obtained through the PCA index (without Bayesian)	93%	85%	86%	21%
ADR Base Dataset + CI + Bayes	It includes the variables of the Base dataset plus the percentage of credibility obtained through the PCA index plus the Bayesian network model inferred between the HGD relationships: DrugSymptomsSickness.	95%	90%	91%	25%

**Table 4:** Benchmark analysis external.

Metric	Random Forest	Deep Learning bi-LSTM	Neural LSTM with CRF	SVM	Decision Tree	This Work DEP-DST
Precision	92.3% [28]	86% [29]	84.5% [30]	90.7% [28]	87.3% [31]	90%
Accuracy	88% [28]	86% [29]	84% [30]	70.6% [28]	87.3% [31]	95%
Recall	85.7% [28]	86% [29]	71.9% [30]	59.9% [28]	87.3% [31]	91%
Number of hypotheses	keeps	keeps	keeps	keeps	keeps	reduces
Degree of Certainty	not changed	not changed	not changed	not changed	not changed	increases
Base used	EHR-Galdakao-Usansolo Hospital	corpus ixaMedGS and ixaMedCH	EHR cancer patients	EHR-Galdakao-Usansolo Hospital	EHR okkaido University Hospita	EHR INCA-Cancer Hospital brazilian
Characterhistics of Base	descriptive high dimensionality	descriptive high dimensionality	descriptive high dimensionality	descriptive high dimensionality	descriptive high dimensionality	descriptive high dimensionality

## Data Availability

Not applicable.
